# Systemic sclerosis triggered by bleomycin: a 5-year follow-up

**DOI:** 10.1016/j.ero.2025.11.012

**Published:** 2025-12-06

**Authors:** Philipp Dittert, Martin Krusche, Lennard Ostendorf, Gerhard Krönke, Udo Schneider, Robert Biesen

**Affiliations:** 1 Department of Rheumatology and Clinical Immunology, Charité, Universitätsmedizin Berlin, Corporate Member of Freie Universität Berlin and Humboldt-Universität zu Berlin, Berlin, Germany; 2 Division of Rheumatology and Systemic Inflammatory Diseases, III, Department of Medicine, University Medical Center Hamburg-Eppendorf, Hamburg, Germany; 3 Department of Nephrology and Medical Intensive Care, Charité, Universitätsmedizin Berlin, Corporate Member of Freie Universität Berlin, Humboldt-Universität zu Berlin, and Berlin Institute of Health, Germany; 4 Deutsches Rheuma Forschungszentrum (DRFZ), an Institute of the Leibniz Association, Berlin, Germany; 5 Division of Rheumatology, Inflammation, Immunity, Brigham and Women’s Hospital, Harvard Medical School, Boston, MA, USA; 6 Department of Rheumatology, Clinical Immunology and Osteology, Immanuel Hospital Berlin-Wannsee Branch, Berlin, Germany

Systemic sclerosis (SSc) is a rare autoimmune disease characterised by vasculopathy, fibrosis, and immune dysregulation. Indirect HEp-2 immunofluorescence (HEp-2 IIF) test is positive in 95% of patients, showing characteristic SSc patterns and corresponding disease-specific autoantibodies. Exposure to exogenous agents, including drugs such as nivolumab or bleomycin, chemicals like vinyl chloride, or toxins such as silicone and those associated with toxic oil syndrome, can trigger SSc-like features — or pseudoscleroderma [1]. Here, we report the induction and persistence of SSc and autoantibodies over a period of 5 years following exposure to bleomycin in a patient.

A previously healthy 27-year-old white European male was diagnosed with a nonseminomatous, mixed germ cell tumour of the testis (pathological TNM classification: pT2 V1 L0 R0). A unilateral orchiectomy was performed. Twelve weeks later, tumour markers — alpha-fetoprotein (AFP) and beta-human chorionic gonadotropin (*ß*-hCG) — increased. Therefore, chemotherapy with bleomycin, etoposide, and cisplatin (BEP) was initiated.

With the first bleomycin application, the patient reported for the first time cold, blue, and painful fingers. A classic Raynaud’s phenomenon developed, which gradually worsened.

Six weeks after the initial administration of chemotherapy, treatment was discontinued due to severe Raynaud’s phenomenon, pitting scars, and bilateral sclerodactyly (Fig, A1), and a rheumatologist was consulted. The examination revealed a positive Prayer sign due to contractures of the proximal interphalangeal joints, skin hyperpigmentation, and digital ulcerations. Modified Rodnan skin score (mRSS) was 6 out of 51. Indirect immunofluorescence on HEp-2 cells revealed an AC-4 pattern at a titer of 1:5120 and a concurrent AC-8 pattern at 1:2560 (Fig, A2). The 13-antigen SSc blot (Euroimmun) showed strong positivity for anti-PM-Scl100 and anticentromere antibodies, and weak reactivity against antiribopolymerase III (RP155). Capillaroscopy showed capillary rarefaction, megacapillaries, and ectasia with repeated microbleeds, indicating an active pattern (Fig, A1 right). High-resolution chest computed tomography (Fig, A3), pulmonary function testing, and echocardiography were unremarkable. In summary, the ACR classification criteria for SSc were fulfilled with a score of 14, exceeding the threshold of 9 [2]. Due to the presence of digital ulcers, treatment for vasculopathy with iloprost infusion was initiated, followed by oral calcium channel blockers. Digital ulcerations healed (Fig, B) and left pitting scars, without the occurrence of new ulcerations. The patient was lost to follow-up.

**Figure:**
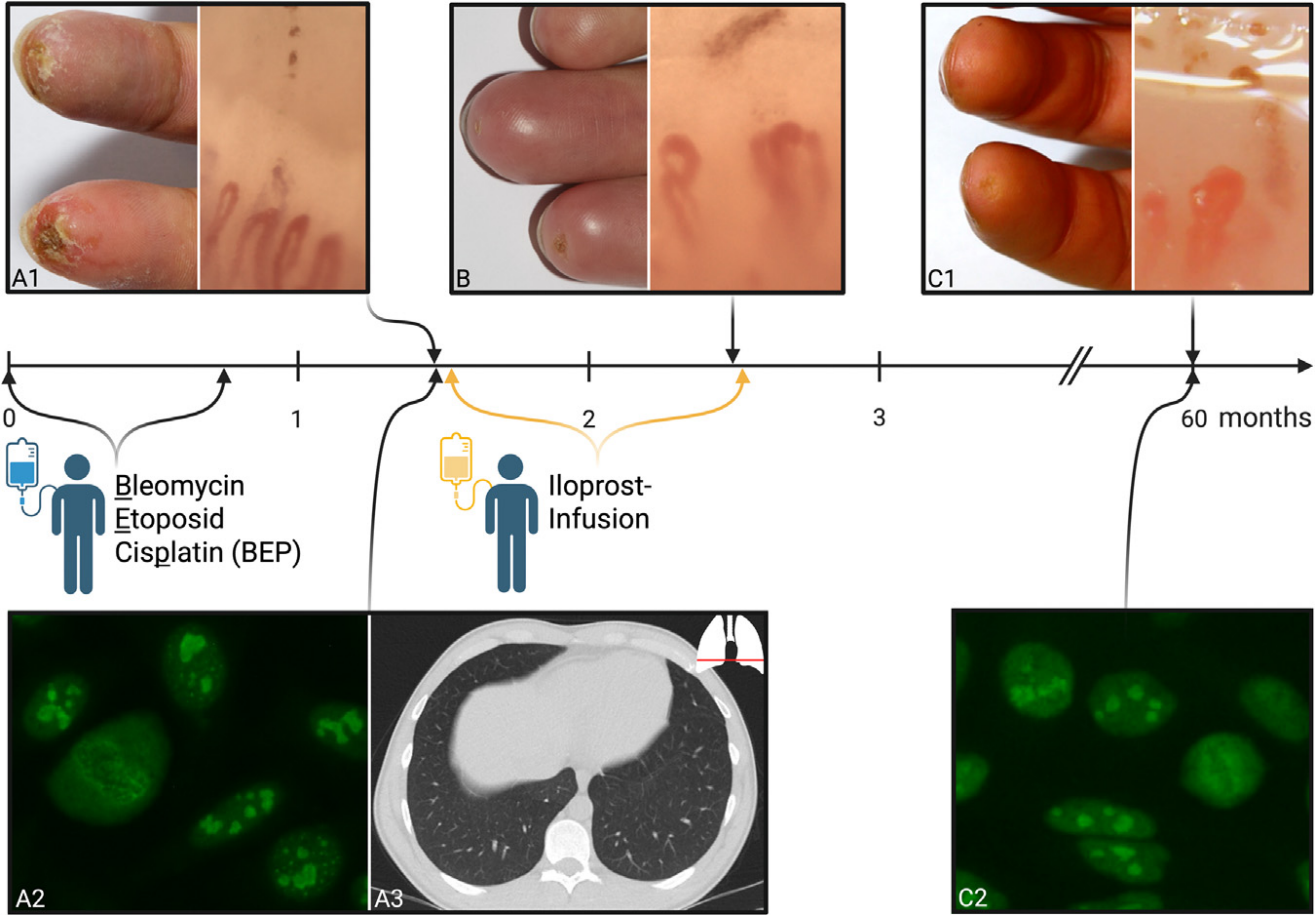
Timeline summarising key findings in bleomycin-induced systemic sclerosis. (A1-A3) Week 6 after the start of BEP (bleomycin, etoposide, and cisplatin) chemotherapy: (A1) Left panel: Severe digital ulcerations. Right panel: Nailfold capillaroscopy showing an active scleroderma pattern. (A2) Indirect immunofluorescence on HEp-2 cells reveals a fine-speckled nuclear pattern (AC-4) and a homogeneous nucleolar pattern (AC-8). (A3) High-resolution chest computed tomography shows no evidence of interstitial lung disease (ILD). (B) Week 10 after BEP: Left panel: Clinical improvement in cutaneous findings following discontinuation of chemotherapy and 4 weeks after iloprost infusion. Right panel: Nailfold capillaroscopy still shows an active scleroderma pattern. (C1, C2) Five years after BEP: (C1) Left panel: Pitted scars are visible without new ulcerations. Right panel: Nailfold capillaroscopy demonstrates stable findings. (C2) HEp-2 indirect immunofluorescence shows a persistent homogeneous nucleolar pattern (AC-8). Figure created with BioRender.com.

Five years later, the patient presented again by chance. Dermatosclerosis had completely remitted (mRSS = 0), and there was again no evidence of further organ involvement. Raynaud’s phenomenon still occurred occasionally, particularly after cold exposure. Capillary microscopy continued to show megacapillaries, ectasia, and haemorrhages consistent with an active pattern (Fig, C1). HEp-2 IIF test revealed an AC-8 pattern at a titer of 1:1280 (Fig, C2). The SSc blot showed strongly positive anti-PM-Scl75 and anti-PM-Scl100 and weakly positive anticentromere B antibodies, whereas antibodies against ribopolymerase III were no longer detectable.

Since its introduction in the 1970s, bleomycin has been linked to rare side effects, including hyperpigmentation, hyperkeratosis, and scleroderma-mimicking cutaneous changes. These findings led to the development of a SSc mouse model that is still important today [3]. Bleomycin induces endothelial and fibroblast-derived cytokine release, leading to vascular damage [4] and fibrosis, and contributes to the development of SSc-specific autoantibodies via B cell activation [5].

A literature review identified 13 cases of bleomycin-induced SSc fulfilling the American College of Rheumatology/European Alliance of Associations for Rheumatology criteria [2]. However, only 4 reported a positive antinuclear antibody titer [6], and none described disease-specific autoantibodies or included long-term follow-up—highlighting the uniqueness of this case with persistent specific autoantibody expression.

The most important differential diagnosis in our case is paraneoplastic SSc [7]. Supplementary Table compares the arguments for bleomycin- and paraneoplastic-induced SSc. The clinical picture suggests drug-induced SSc due to the acute and limited course, young age, mainly cutaneous involvement, the presence of several SSc-specific autoantibodies, and good prognosis after discontinuation of bleomycin without any SSc-specific therapy.

This case illustrates how short-term exposure to exogenous agents such as bleomycin in susceptible individuals can lead to clinical features of SSc and disease-specific autoantibodies, which persist for years even after elimination of the trigger.

## Appendix Supplementary materials

Image, application 1

## Footnotes

Supplementary material associated with this article can be found in the online version at doi:10.1016/j.ero.2025.11.012.
