# Supplementary material for: Systemic sclerosis triggered by bleomycin: a 5-year follow-up
Source: EULAR Rheumatol Open. 2025 Dec 6;1(4):462–4. doi: 10.1016/j.ero.2025.11.012 (PMC13292139; doi:10.1016/j.ero.2025.11.012)
Supplement: Supplementary file 1 [file mmc1.docx]

**Supplementary Table**: Paraneoplastic versus drug induced systemic sclerosis

| **Aspect** | **Paraneoplastic SSc** | **Drug-Induced SSc** |
| --- | --- | --- |
| **Temporal relationship** | ✅ Close temporal association with tumour diagnosis or therapy (usually within ±2 years of SSc onset) | ✅ In drug-induced forms, onset occurs shortly after exposure |
| **Age** | ❌ Typically older patients (>50 years) | ✅ Often younger, especially in drug-induced cases |
| **Clinical onset and course** | ❌ Abrupt onset, rapidly progressive, usually diffuse cutaneous subtype | ✅ Acute, but often limited and milder, frequently reversible |
| **Organ involvement** | ❌ Common: interstitial lung disease (ILD), cardiac involvement, scleroderma renal crisis | ✅ Usually cutaneous only, here fitting well to bleomycin |
| **Autoantibody profile** | ❌ Typically anti–RNA polymerase III or anti-Scl70 positive; no multiple SSc-Ab´s | ⚖️ Usually unspecific nucleolar ANA |
| **Tumour types** | ❌ Most often breast, lung, GI or gynaecologic cancers | ✅ Testicular germ cell tumour is not associated to SSc. |
| **Response to therapy** | ⚖️ Improves only with successful cancer treatment; poor response to immunosuppression alone | ✅ Improves after discontinuation of the triggering drug; immunosuppression usually limited or unnecessary |
| **Prognosis** | ❌ Often poor due to rapid progression and underlying malignancy | ✅ Good if the trigger is removed early; typically non-progressive |

Pro (✅ ), balanced (⚖️) and contra(❌) arguments
